# A Bibliometric Analysis of Research Progress and Trends on Fly Ash-Based Geopolymer

**DOI:** 10.3390/ma15144777

**Published:** 2022-07-07

**Authors:** Quanzhi Tian, Yinhai Pan, Yingchu Bai, Shuo Yao, Shiqiang Sun

**Affiliations:** 1 National Engineering Research Center of Coal Preparation and Purification, China University of Mining and Technology, Xuzhou 221116, China; tianqz0502@cumt.edu.cn (Q.T.); ts20040089a31@cumt.edu.cn (Y.P.); byc13111202118@163.com (Y.B.); 15150010836@163.com (S.Y.); 2 School of Chemical Engineering and Technology, China University of Mining and Technology, Xuzhou 221116, China; 3 School of Environmental Science and Engineering, Tongji University, Shanghai 200092, China

**Keywords:** geopolymer, fly ash, citespace, visualization analysis

## Abstract

The objective of this work is to present the research progress and applications of fly ash-based geopolymer, and summarize the future research hotpots. Since 1998, scholars have made important contributions to the study of fly ash-based geopolymer, and a large number of research studies have been published. Therefore, a bibliometric analysis for the determination of the research status, trend, and history of fly ash-based geopolymer was conducted in the present study. A total of 4352 publications on fly ash-based geopolymer were collected between 1998 and 2022, with an increasing trend year by year. China and Australia are the largest contributors to the field, and the research institutions in each country cooperate closely. In addition, the most contributing research areas are MATERIALS SCIENCE, ENGINEERING, and CONSTRUCTION & BUILDING TECHNOLOGY. The keywords including fly ash, compressive strength, and mechanical property are the most frequently appearing words. On the whole, the development of fly ash-based geopolymer could be divided into three stages including the replacement of ordinary Portland cement, the development of multifunctional materials, and the reduction of environmental impact by the conversion of solid waste. This overview could provide an important guidance for the development of fly ash-based geopolymer.

## 1. Introduction

The large-scale production of conventional Portland cement accounts for approximately 7% of total CO_2_ emissions globally [1]. To control CO_2_ emissions, geopolymer is currently a research hotspot as a potential substitute for ordinary Portland cement because of its lower CO_2_ emission and excellent properties such as higher mechanical strength, better heat resistance, excellent resistance to acid, sulfate, etc. [2,3]. Geopolymer is a kind of inorganic binder consisting of aluminum and silicon which are polymerized under alkaline condition at a low temperature. It has been verified that solid wastes including fly ashes, slags, mining tailings, and metallurgical wastes have been studied as the source materials for geopolymer synthesis [4,5]. Among them, fly ash is the most widely used as raw material for the preparation of geopolymer. It is well known that fly ash as an industrial waste can replace cement for construction due to its pozzolanic activity. Specifically, the active SiO_2_ and Al_2_O_3_ in fly ash can dissolve and participate with calcium to form calcium silicate hydrate and calcium aluminate hydrate [6]. Similarly, these dissolved SiO_2_ and Al_2_O_3_ species can be polymerized under alkaline condition with less calcium, contributing to the formation of geopolymer. For decades, the research related to fly ash-based geopolymer has become a multidisciplinary field, covering a wide variety of aspects including concrete [7], solidification material [8], adsorbent [9], thermal insulation material [10], etc. On the other hand, a large amount of fly ash is produced annually in the world. Serious contamination can be caused to air, water, and land if fly ash is unproperly disposed of. Therefore, it is also urgent to develop new technologies for the treatment of fly ash. As stated above, the preparation of geopolymer can be an effective way to the recycling utilization of fly ash, and the preparation of fly ash-based geopolymer could achieve multi-benefits. To get a better understanding of fly ash-based geopolymer, a summary of the research status is required. Even though several scientific reviews on fly ash-based geopolymer have been published, it is urgent to portray the research progress and global trend on fly ash-based geopolymer from a view of bibliometrics.

Specifically, bibliometric is a kind of research methodology involved in the library and information science, which has been widely used to analyze research performance and assess the research trends through investigating the publication characteristics such as authorship, source, subject, and citation. [11]. Yang et al. [12] analyzed the retrieved data and determined the most frequently used keywords, the most referenced authors and papers, and the locations with the most publications on the topic of geopolymer composites. Krishna et al. [13] analyzed the current research trend of graphene reinforced geopolymers by bibliometric analysis. However, there have been few reports about bibliometric analysis of fly ash-based geopolymer to date. This is not beneficial for the development of fly ash-based geopolymer with high quality. Therefore, based on the bibliometric and visual analysis, the research progress of fly ash-based geopolymer from 1998 to 2022 was reviewed in the present study. The research hotspots in this field were identified and their most popular application areas were summarized. Besides, the characteristics and development trends of fly ash-based geopolymer were highlighted, and future research directions were foreseen.

## 2. Fly Ash-Based Geopolymer

Fly ash is a kind of solid residue produced from the combustion of coal or other wastes. Due to the rapid cooling process, the generated fly ash always contains a certain amount of amorphous substance which typically contains a large amount of SiO_2_ and Al_2_O_3_ [14]. The amorphous substance can be dissolved in alkaline condition. This is the main reason why fly ash could be used as raw material for the synthesis of geopolymer. The basic principle of the formation of fly ash-based geopolymer is the alkali-activated dissolution of aluminosilicate in fly ash [15]. However, the activated reaction process is extremely complicated. Generally, the silicate and aluminate in fly ash can be transformed into a soluble state under alkaline condition. Then, these soluble species would be condensed through the formation of polysialate –Al–O–Si– chains. Specifically, the active silicate and aluminate species react to form nuclei and oligomer, followed by the formation of a three-dimensional structure [16]. There are several factors influencing the final structure of the generated geopolymer. Firstly, the Si/Al molar ratio of active substance in fly ash could have a remarkable effect on the geopolymer properties such as mechanical strength, porosity, etc. Excessive higher or lower values of Si/Al molar ratio are not beneficial for the preparation of fly ash-based geopolymer [17]. In addition to the Si/Al molar ratio, the alkaline solution can be another important factor in the formation of fly ash-based geopolymer. For example, the K^+^-based activator can contribute to a lower surface charge density and a higher degree of polymerization of the geopolymer matrix, due to the larger radius of cation K^+^ (1.33 Å) than the Na^+^ cation (0.97 Å) [17]. In addition, it should be noted that the dissolution of fly ash is not completed at room temperature, thereby increasing the setting time of fly ash-based geopolymer. Thus, higher temperature curing is normally necessary. As a whole, even though there have been many studies about fly ash-based geopolymer, the research status and progress should be clarified to guide the future study.

## 3. Method and Data Source

CiteSpace is a Java-based scientific visualization and measurement software developed by Professor Chaomei Chen [18]. It can utilize the extensive documentation of a research area to map the knowledge developments in science and technology, generating an overview of the research area [19]. By qualifying modules such as country, author, keywords, and cited information, it can selectively visualize and analyze scientific issues, presenting the panorama of information in the field of scientific knowledge and identifying the research base, key literature, research hotspots, and frontier directions in a scientific field [20].

To ensure the completeness, accuracy, and high reliability of the original data, the Science Citation Index Extension (SCIE) module of the Web of Science Core Database (WosCC), which had excellent adaptability to CiteSpace software, was chosen as the data source for this study. According to the search rules, the search formula was set as TS (Topic Search) = ((geopolymer) AND (fly ash)). Based on the search results, the first study on fly ash-based geopolymer in English was found to be completed in 1998 [21]. Therefore, the search period for this study was set as 1998–2022. After excluding non-academic papers such as editorial material and conference abstracts, a total of 4352 documents were obtained. CiteSpace (5.8 R3) software was used to analyze and process the collected literature data, and the node types of the study included author, institution, country, keyword, source, category, citation information, etc.

## 4. Results and Discussion

### 4.1. Publication Characteristics

**Figure 1:**
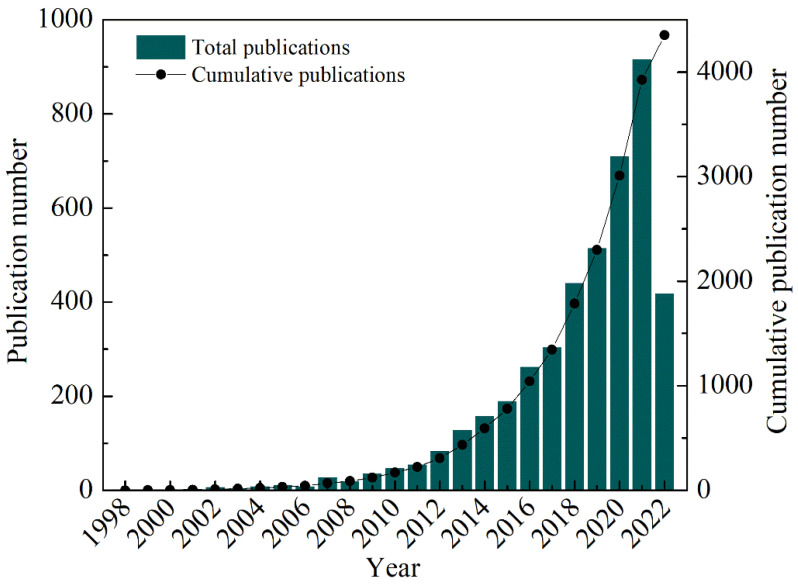
Distribution of scientific publications on fly ash-based geopolymer in 1998–2022.

By screening and de-duplicating publications on fly ash-based geopolymer in the WoSCC database, the obtained 4352 publications consisted of two main types including research article and review. The proportion of research articles was 93.61%, and accordingly, review papers account for 6.39%. Figure 1 showed the annual and cumulative number of relevant publications about fly ash-based geopolymer for the period from 1998 to 2022. There are few studies on fly ash-based geopolymer reported in the WoS database between 1998 and 2011. However, from 2012 onwards, the number of publications in this research area significantly increased, and their number has increased by more than 50% compared to 2011 (Table S1). This is mainly because fly ash as a kind of solid waste has gradually attracted the attention of researchers from 1998. With the release of government policies on the environment, it is imperative to develop new technologies for the treatment of fly ash. Since then, the total and the cumulative number of publications on fly ash-based geopolymer has increased each year and reached a peak in 2021. The number of relevant papers in 2021 was 915, accounting for 21.02% of the total literature for this study. It should be noted that due to the period of this study ending in June 2022, the number of publications in 2022 is lower than that in 2021. The preparation of geopolymer using industrial fly ash as raw material not only enables the reduction and reuse of solid waste, but also saves energy and resources, and reduces environmental pollution. Thus, it has consistently received increasing attention over the years and the number of relevant scientific studies and papers increases significantly.

### 4.2. Country Contributions

**Figure 2:**
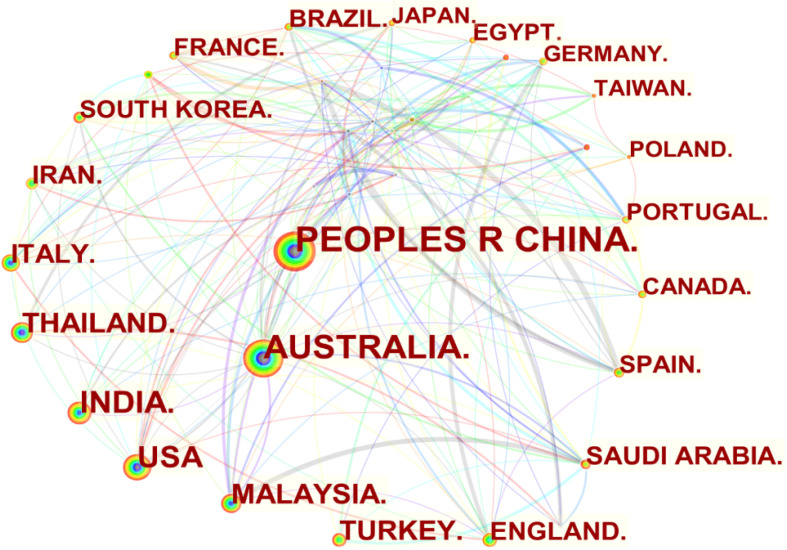
Visualisation of country contributions in the field of fly ash-based geopolymer in 1998–2022.

Based on CiteSpace analysis, 117 countries have carried out scientific research on fly ash-based geopolymer from 1998 to 2022. The visualization of the countries’ contributions are shown in Figure 2 where the larger the node, the more publications the corresponding country has produced. The connecting lines between the nodes indicate the network of collaborative relationships between countries. It can be seen from Table 1 that China had 983 publications on fly ash-based polymers in English in the WoS database, accounting for 16.45% of all countries during the period 1998–2022. Australia came in second place with 606 papers, accounting for 10.14% of the total number of published papers. Both China and Australia maintain collaborative relationships with a wide range of countries, indicating that both China and Australia have a strong research base and a good network of international cooperation in the field of fly ash-based geopolymer and have contributed greatly to the continued progress in this field. India and United States accounted for 7.67% and 6.68% of publications in this field, respectively, ranking third and fourth. From a regional perspective, the joint efforts of China, India, Malaysia, Thailand, Turkey, and Saudi Arabia have made Asia a world leader in the achievement of fly ash-based geopolymer. By contrast, Europe and America are the ones contributing relatively little to the field, which is mainly related to the differences in the energy mix in the different global regions. Asian countries are dominated by coal in their energy consumption, while European countries have the highest share of renewable energy consumption and the Americas have natural gas as the dominant source of energy [22].

The rapid development of fly ash-based geopolymer is mainly due to the countries’ energy structures. China is the world’s largest producer and consumer of coal. By 2025, the annual comprehensive production capacity of domestic energy will reach over 4.6 billion tons of standard coal [23], indicating that coal would still be the main source of energy in China. Fly ash as an inevitable solid waste from coal combustion was produced in the amount of 650 million tons in 2020, with a total storage of over 3 billion tons in China [24]. China’s contribution to the field of fly ash-based geopolymer is inextricably linked to policy and legal support. To effectively monitor and manage the comprehensive utilization of fly ash, the administrative measures for the comprehensive utilization of fly ash were introduced in 1994 and then revised in 2013 [25]. Specifically, the measures stipulate that the government fully supports the development of technologically mature new wall materials with a large blending capacity of fly ash, and meanwhile encourages the use of fly ash as a cement mix, a substitute for clay in raw materials for batching, etc. To continuously improve and expand the technical level for the comprehensive utilization of bulk solid waste with emphasis on fly ash, the National Development and Reform Commission issued a notice on promoting industrial agglomeration and development of comprehensive utilization of bulk solid waste in 2019 [26]. It emphasizes the need to develop large-mix fly ash concrete technology, product quality of fly ash blocks, and other new building materials, and to continue to expand the scale of application in the field of building materials.

### 4.3. Author Contribution

**Figure 3:**
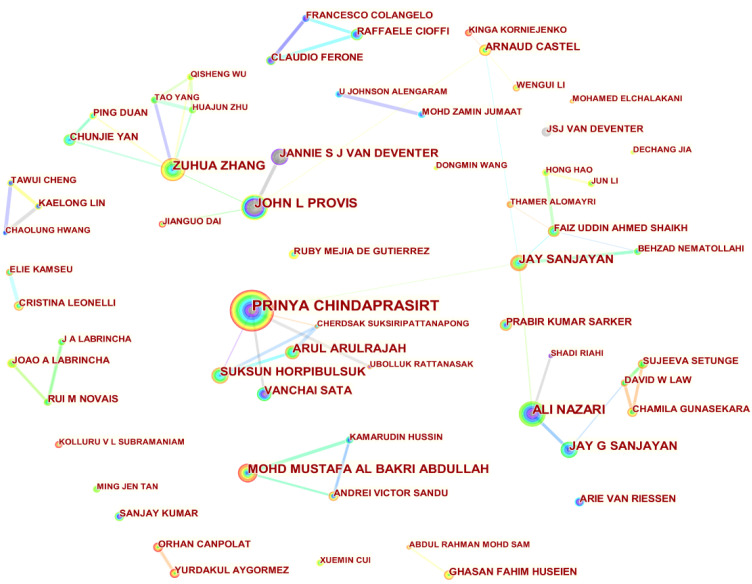
Visualisation of a collaborative network of authors in the field of fly ash-based geopolymer.

This part of the visual analysis can reveal the researchers who have made outstanding contributions to the study of fly ash-based geopolymer and the collaboration between them. By selecting the Author node in the CiteSpace software, a total of 820 academics were found to have conducted related works from 1998 to 2022. As shown in Figure 3 and Table 2, Professor Prinya Chindaprasirt of Khon Kaen University in Thailand is in first place with 96 scientific papers in the field of fly ash-based geopolymer, accounting for 2.56% of the total number of papers. He also works closely with several authors, including Professor Suksun Horpibulsuk (ranking ninth in Table 2) of Suranaree University of Technology, and Professor Arul Arulrajah (ranking eighth in Table 2) of Swinburne University of Technology. Professor Ali Nazari from Islamic Azad University and Professor Mohd Mustafa Al Bakri Abdullah from Universiti Malaysia Perlis ranked second and third with 59 (shared 1.57%) and 56 (shared 1.49%) papers, respectively. Professor Ali Nazari collaborates more closely with Professor Jay Sanjayan of the Swinburne University of Technology (in sixth place). Professor John L Provis has a close working relationship with Professor Zuhua Zhang of Hunan University in fourth place and Professor Jannie S J Van Deventer of the University of Melbourne in ninth place. The top ten authors from different countries are collaborating to promote the field of fly ash-based geopolymer. It is worth mentioning that Professor Zuhua Zhang of Hunan University, with 55 publications (1.47%), is the only Chinese scholar in the top 10 and maintains a close network of collaboration with many other Chinese authors.

### 4.4. Institution Cooperation

**Figure 4:**
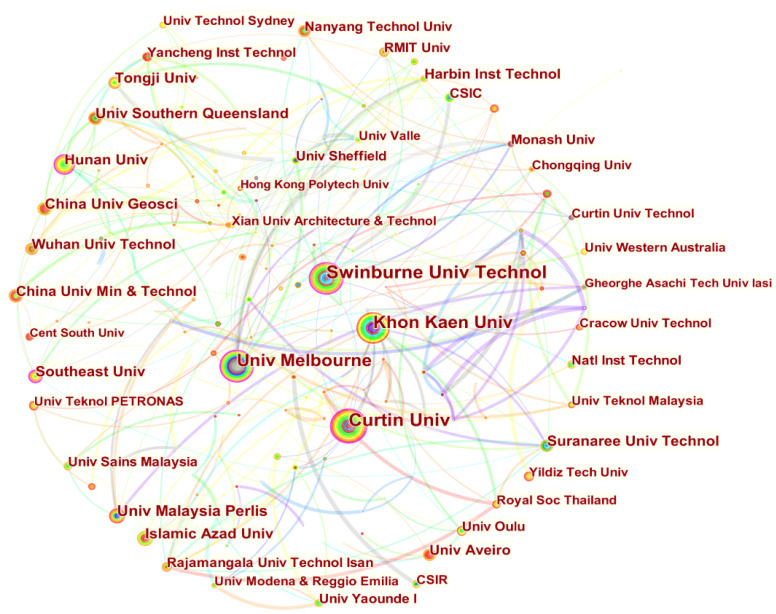
Visualisation of a collaborative network of institutions in the field of fly ash-based geopolymer.

This section aims to visualize the distribution of the main research institutes and universities in the field of fly ash-based geopolymer and to give a clear picture of the cooperation between the various institutions. By selecting the Institution node in the CiteSpace software, the resulting institutional collaboration diagram is shown in Figure 4 where the size of the points represents the size of the institution’s contribution to the field and the thickness of the line represents the degree of collaboration between institutions. It can be seen that the Swinburne University of Technology ranked first in the number of publications with 126, representing 2.98% of the total publications (Table 3). Curtin University ranked second with 119 publications, and the University of Melbourne ranked third with a contribution of 2.60%, and Khon Kaen University was fourth with a contribution of 2.48%. Figure 4 also shows that all four of these institutions maintain strong collaborative relationships with other institutions. Notably, Hunan University, China University of Geosciences, and Tongji University from China ranked sixth, eighth, and tenth respectively. It can also be seen that the China University of Mining and Technology, Wuhan University of Technology, Harbin Institute of Technology, Yancheng Institute of Technology, etc. have made certain contributions in this field. In addition, four of the top ten institutions are from Australia, which is consistent with the findings in the Section 4.2. More and more institutions have joined the field of fly ash-based geopolymer for scientific research. Meanwhile, the cooperation and competition between institutions would stimulate the continuous innovation of technology and promote the industrial development of fly ash-based geopolymer.

### 4.5. Keywords Analysis

**Figure 5:**
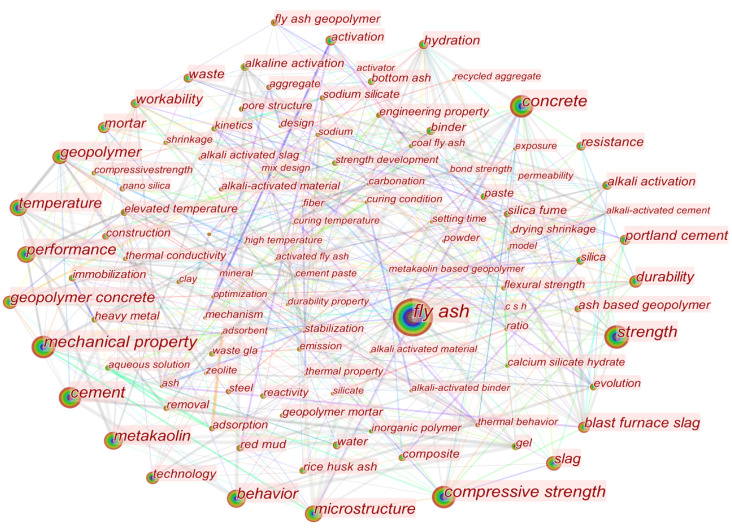
Visualisation of keywords co-occurrence in the field of fly ash-based geopolymer.

Keyword co-occurrence analysis can effectively identify research hotspots and is an indispensable element of scientometric visualization. By selecting the Keywords node in the CiteSpace software, a total of 822 keywords for fly ash-based geopolymer were identified from 1998 to 2022. The keyword co-occurrence relationships in this area are shown in Figure 5, and the top 20 high-frequency, high-centric keywords are shown in Table 4. In Figure 5, the node size represents the occurrence frequency of keywords. The larger node represents that the keyword appears frequently and attracts more attention. Nodes with high centrality are ‘bridges’ to communicate with other nodes, acting as mediators [27]. Therefore, the nodes with both high frequency and centrality are considered to be a hot topic of research in the field of fly ash-based geopolymer [28].

As can be seen, the keyword ‘fly ash’ corresponds to the largest node, with 2689 occurrences over the period 1998–2022. In addition to the high number of studies generated around this keyword, another reason is also related to the search formula set up for this study. The keywords ‘mechanical property’ and ‘compressive strength’ are in the second and third positions with the occurrences of 1191 and 1153, respectively, corresponding to the properties of fly ash-based geopolymer. The keywords ‘concrete’ and ‘cement’, in the fourth and sixth positions, respectively, correspond to the specific application field of this material. As can be seen from the keyword co-occurrence, the studies on fly ash-based geopolymer were mainly focused on the replacement of ordinary Portland cement and concrete for the utilization in construction materials. The reason for this phenomenon is closely related to the compositions and properties of fly ash-based geopolymer, which possess excellent properties such as higher compressive strength, and better durability [29]. On the other hand, to enhance the mechanical and chemical properties of geopolymer, many scholars mixed natural resource minerals (e.g., metakaolin) with solid wastes (e.g., fly ash, blast furnace slag) as precursor materials for geopolymer [30]. The high-frequency keywords reflect the research hot spots of fly ash-based geopolymer, and the multiple symbiotic networks formed by these keywords also reflect the interconnections between the keywords and key technologies.

Figure S1 shows the top 20 keywords with the strongest citation bursts. As can be seen, ‘temperature’ is the most strongly cited keyword in the field, with a strength of 22.87. This is because the temperature has a very significant effect on the compressive strength of fly ash-based geopolymer. Within a certain temperature range, the compressive strength increases as the curing temperature rises. However, a continued rise in temperature would lead to cracks on its surface, leading to a decrease in compressive strength [31]. The occurrence of strongly cited keywords ‘gel’ and ‘inorganic polymer’ revealed the physical and chemical properties of fly ash-based geopolymer, while the occurrence of the keywords ‘technology’ and ‘alkali activation’ indicated their more sought-after synthesis techniques. Depending on the activation pathway of the aluminosilicate precursor, geopolymer synthesis techniques can be divided into alkali-activated and acid-activated polymerization techniques, of which alkali-activated polymerization is the dominant [32,33].

**Figure 6:**
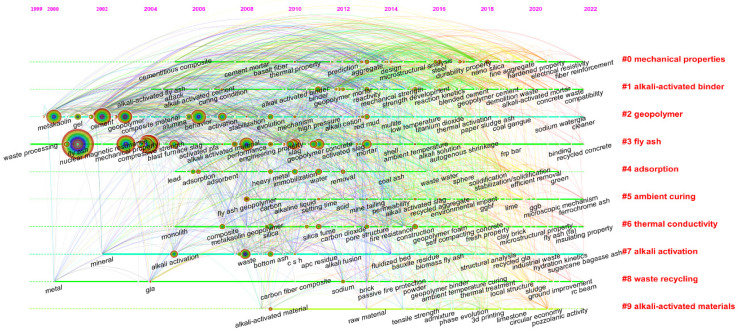
Timeline map of keywords in terms of fly ash-based geopolymer.

The timeline chart reflects the evolution of keywords over time, visually showing the research hotspots and their trends at different periods. Figure 6 shows the timeline map for the 10 clusters in the fly ash-based geopolymer field from 1998 to 2022. As can be seen, although the period begins in 1998, the keywords in the timeline graph appeared in 1999. Research in recent years has focused on the properties (e.g., #0 and #6 cluster), synthetic methods (e.g., #1, #5, #7 and #9 cluster), applications and implications (e.g., #4 and #8 cluster) of fly ash-based geopolymer. The most frequently occurring keywords between 2000 and 2006 were ‘fly ash’, ‘mechanical property’, ‘compressive strength’, and ‘cement’. Since 2006, the keywords ‘adsorption’, ‘heavy metal’, ‘immobilization’, ‘water’, ‘removal’, and ‘fire resistance’ appeared. This reveals that the studies on fly ash-based geopolymer are no longer limited to the application in building materials, but also focus on the adsorption and removal of heavy metals from wastewater, the immobilization of heavy metals, and the study of adiabatic, fireproof and insulating materials. Therefore, the year 2006 can be seen as a milestone in the field of fly ash-based geopolymer. As research continues, scholars began to explore the use of more solid wastes (e.g., tailings, red mud, coal gangue, slag, etc.) as precursors for the preparation of geopolymers to achieve waste recycling. The emergence of ‘environmental impact’ indicates that the research focus in this field has gradually evolved from technological innovation to environmental and ecological impacts. The emergence of ‘circular economy’ in 2019 confirms these conclusions and suggests that the research in this field would be changed in line with national policy directions. As a whole, more and more researchers begin to focus on the industrial application of fly ash-based geopolymer. Therefore, the development of fly ash-based geopolymer can be summarized in three stages: (1) focus on their mechanical properties as a replacement for ordinary Portland cement; (2) development of multi-functional geopolymer-based materials; (3) synergistic disposal of solid wastes for the preparation of geopolymers and reduced environmental impact.

### 4.6. Category Analysis

**Figure 7:**
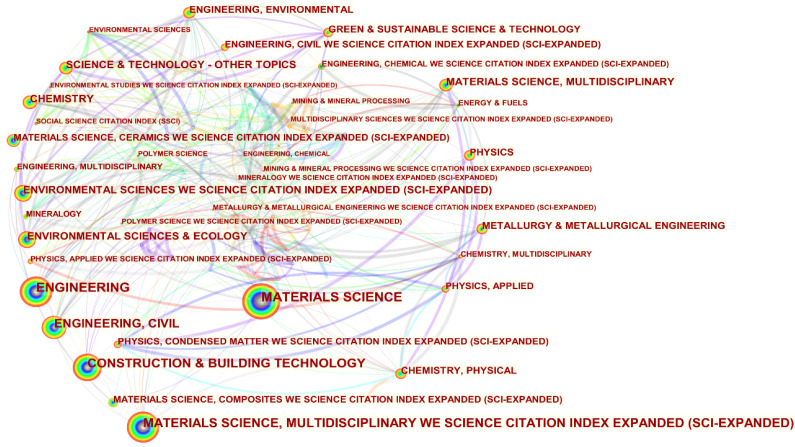
Visualisation of category co-occurrence in the field of fly ash-based geopolymer.

The purpose of the category visualization analysis is to clarify the classification of categories within the field of study and to understand the relationships between the categories. A visualization of the category co-occurrence and a list of the top 20 categories in the field of fly ash-based geopolymer are shown in Figure 7 and Table S2, respectively. Of the 4352 scientific publications collected, 126 categories were involved in the field, with the category ‘MATERIALS SCIENCE’ having the largest node with a contribution of 16.04%. The category “ENGINEERING” with a contribution of 13.39% and “CONSTRUCTION & BUILDING TECHNOLOGY” with a contribution of 10.50% were ranked second and third, respectively. It is worth noting that five of the top 20 contributing categories are linked to the field of ‘materials’ due to the nature and application of fly ash-based geopolymer [30,33]. The high number of its applications also determines that two of the top 20 categories are linked to the field of ‘civil engineering and construction’. In addition, four categories are linked to the field of ‘environment’ because geopolymers can be prepared from solid wastes such as fly ash, red mud, and slag which not only occupy a large number of land resources but also inevitably pose a great threat to the natural environment [34]. Besides, fly ash-based geopolymer also play an important role in the ‘environmental’ discipline as they can be used for the solidification of heavy metals and the adsorption of harmful pollutants from wastewater [35,36,37].

### 4.7. Author Co-Citation Analysis

**Figure 8:**
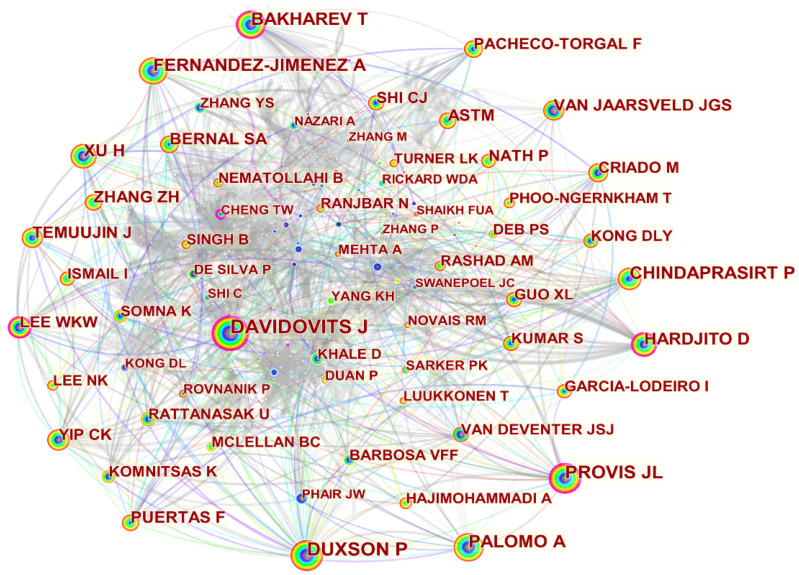
Visualisation of author co-citation in the field of fly ash-based geopolymer.

The purpose of this section is to capture the high-impact authors in the field and the collaborations between these authors. The Cited author node was selected in CiteSpace software and the top 50 cited authors between 1998 and 2022 were analyzed, as shown in Figure 8. It can be seen that Professor Davidovits J of France is the most cited author with 2203 citations and a contribution of 6.82%, ranking first in terms of author co-citations (Table S3). In 1978, Professor Davidovits J discovered and named this kind of geopolymer material with a three-dimensional structure [38]. He went on to further investigate the types of geopolymers and their polymerization mechanisms, leading the way in the development of geopolymers [39]. Professor Duxson P from Australia ranked second with 1926 citations and Professor Provis JL from the University of Melbourne ranked third with a contribution of 3.51%. Moreover, citations to publications by the above three academics in the scientific literature in the field of fly ash-based geopolymer form a collaborative network, indicating that their scientific research is strongly recognized among their peers. These three scholars and their teams have played an integral role in this research area, advancing the theory and technology in the field of fly ash-based geopolymer.

### 4.8. Journal Analysis

**Figure 9:**
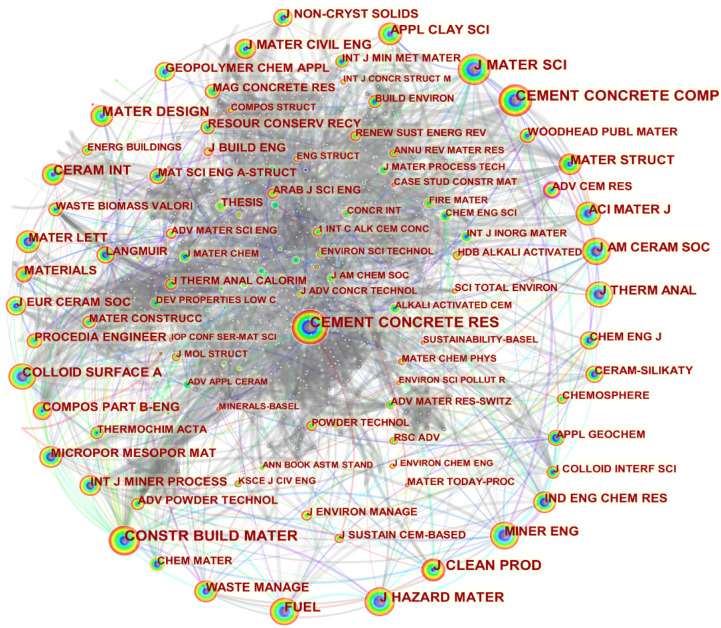
Visualisation of journal co-citation in the field of fly ash-based geopolymer.

A visual analysis of the mainstream cited journals in the field of fly ash-based geopolymer was carried out and the results are shown in Figure 9 and Table S4 respectively. The journal CONSTR BUILD MATER was the most cited, with 3654 citations. The journals CEMENT CONCRETE RES and CEMENT CONCRETE COMP are in second and third place with 3565 and 3002 citations, respectively. As can be seen from the top three most-cited journals, it is verified again that the studies on fly ash-based geopolymer have focused on replacing ordinary Portland cement and concrete as a new green and sustainable building material. The mass production of ordinary Portland cement not only releases a large amount of CO_2_ but also consumes mineral resources and non-renewable energy in quantities. In addition, the preparation of geopolymers from solid waste such as fly ash has received much attention, driving the journal of J CLEAN PROD to rank fourth with 2251 citations. The journal J HAZARD MATER is also highly cited with 1872 citations ranking fifth, based on the research into the solidification and adsorption of toxic and hazardous substances such as heavy metals by fly ash-based geopolymer. Figure S2 shows the top 25 cited journals with the strongest burst. The top-ranked journal, MINER ENG, began to emerge in 1999 and ended in 2015 with an intensity of 61.14. This is because some by-products from the mining industry such as coal gangue can be used as raw material for the preparation of geopolymers, thus driving the citation emergence of MINER ENG in the field of fly ash-based geopolymer. According to the analysis of the top 20 cited journals in this field, the journals with high citation frequency mainly have the following characteristics: (1) design, structure, and application of building materials such as cement, concrete, and ceramic materials, (2) clean production, (3) hazardous waste disposal technology (4) mineral engineering, (5) colloidal surface chemistry.

### 4.9. Document Analysis

**Figure 10:**
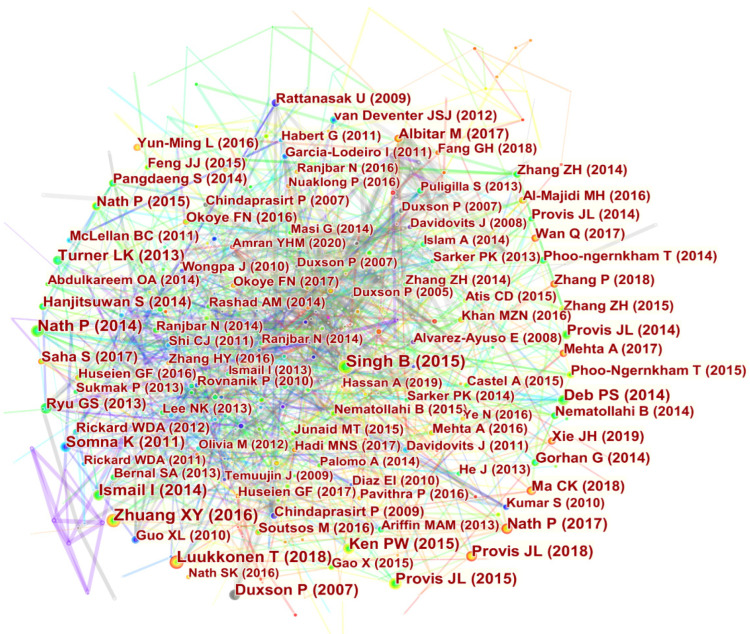
Visualisation of document co-citation in the field of fly ash-based geopolymer.

Co-citation of documents refers to the fact that two documents appear together in a third cited bibliography and it can reflect the close relationship between the published documents. Figure 10 and Table S5 show the collaboration diagram and ranking of cited references in the field of fly ash-based geopolymer, respectively. The documents with all of the highest number of co-citations are centered in the last decade, indicating that the field of fly ash-based geopolymer has been attracted increasing attention over this period, which is consistent with the results of the Section 4.1. The original review article ‘Fly ash-based geopolymer: clean production, properties, and applications’ by Professor Zhuang XY of the Zhejiang University of Technology in the journal J CLEAN PROD was cited 182 times and ranked first. The review ‘Geopolymer concrete: A review of some recent developments’ by Professor Singh B from CSIR-Central Building Research Institute has been cited 181 times and ranked second. It is the only paper published by a Chinese scholar in the top 20 cited references. Luukkonen T’s article ‘One-part alkali-activated materials: A review’ was cited 174 times and ranked third. It is noteworthy that the top three cited papers are all review articles. In addition, Professor Nath P from Curtin University and Professor Provis JL from the University of Sheffield had three articles with high citations in the top 20 cited references. Interestingly, in conjunction with the Section 4.8, most of the top 20 citations were published in highly cited journals such as CONSTR BUILD MATER, J CLEAN PROD, and CEMENT CONCRETE RES. In these cited references, studies on the mechanical property of fly ash-based geopolymer are predominant [40,41,42]. Besides, there are studies comparing CO_2_ emissions during the preparation of fly ash-based geopolymer with ordinary Portland cement and comparing the mechanical properties between them [43,44].

## 5. Findings and Perspective

A scientometric analysis was conducted on published literature of fly ash-based geopolymer to identify the publication characteristics such as country contribution, author contribution, institution cooperation, and keyword analysis. Generally, it was noticed that the promotion of fly ash-based geopolymer still needs substantial exploration for their applicability in various applications on a large scale, although a large number of studies have been conducted. There is still a gap between research, application, and implementation. Firstly, the properties of fly ash for the preparation of geopolymer can fluctuate greatly, leading to a wide variation in the performance of the obtained products and making it difficult to keep them in a stable state. Therefore, standard specifications and test methods should be specifically designed for geopolymer. Secondly, the current preparation of geopolymer requires the addition of high doses of alkali activator, increasing their cost. Future research should focus on the optimization of activator dosage and the development of new activation methods. Complementary and synergistic utilization of various solid wastes should be conducted to obtain better-performing geopolymers. Multifunctional geopolymer-based materials can also be developed from solid wastes with different properties for various applications such as construction materials, road materials, heavy metal curing, and wastewater treatment, depending on the actual engineering needs.

## 6. Conclusions

The production of geopolymer using fly ash can not only provide an effective way for the treatment of industrial solid waste but also reduce the consumption of natural resources and achieve sustainable development. In the present work, a bibliometric analysis of fly ash-based geopolymer was carried out to reveal its research progress and future development tendency. A total of 4352 publications on fly ash-based geopolymer were included in the analysis of this study. China plays an important role in the research of fly ash-based geopolymer, accounting for 16.45% of total publications. Australia came in second place accounting for 10.14%. As for the specific research institutions, Swinburne University of Technology, Curtin University, and the University of Melbourne are early starters in the field of fly ash-based geopolymer and continue to innovate technologies to drive industrial development. Professor Davidovits J of France, Professor Duxson P of Australia, and Professor PROVIS JL of the University of Melbourne are the most cited scholars in this field. On the other hand, the most prominent categories for fly ash-based geopolymer are MATERIALS SCIENCE, ENGINEERING, and CONSTRUCTION & BUILDING TECHNOLOGY. ‘Fly ash’, ‘compressive strength’, and ‘mechanical property’ are three keywords which appear most frequently. As a whole, the development of the fly ash-based geopolymer field can be divided into three stages: (1) mechanical properties to replace ordinary silicate cement; (2) development of multifunctional ground polymer materials; and (3) synergistic use of multiple solid wastes to reduce environmental impact.

## Figures and Tables

**Table 1:** Top 10 countries in terms of contribution to scientific publications.

Ranking	Country	Count	Centrality	Contribution (%)
1	People’s R China (mainland)	983	0.04	16.45
2	Australia	606	0.17	10.14
3	India	458	0.08	7.67
4	USA	399	0.28	6.68
5	Malaysia	259	0.14	4.34
6	Thailand	210	0.01	3.52
7	Turkey	191	0.03	3.20
8	Italy	167	0.1	2.80
9	Saudi Arabia	160	0.07	2.68
10	England	145	0.03	2.43

**Table 2:** Top 10 authors in terms of contribution to scientific publications.

Ranking	Author	Count	Centrality	Contribution (%)
1	Prinya Chindaprasirt	96	0.03	2.56
2	Ali Nazari	59	0.02	1.57
3	Mohd Mustafa Al Bakri Ab-dullah	56	0	1.49
4	Zuhua Zhang	55	0.03	1.47
5	John L Provis	52	0.07	1.39
6	Jay G Sanjayan	36	0.09	0.96
7	Arul Arulrajah	36	0	0.96
8	Suksun Horpibulsuk	35	0	0.93
9	Jannie S J Van Deventer	29	0.01	0.77
10	Vanch Ai Sata	28	0	0.75

**Table 3:** Top 10 institutions in terms of contribution to scientific publications.

Ranking	Institutions	Count	Centrality	Contribution (%)
1	Swinburne University of Technology	126	0.18	2.98
2	Curtin University	119	0.16	2.81
3	The University of Melbourne	110	0.16	2.60
4	Khon Kaen University	105	0.06	2.48
5	University of Malaysia Perlis	68	0.05	1.61
6	Hunan University	62	0.1	1.47
7	University of Southern Queensland	52	0.04	1.23
8	China University of Geosciences	48	0.04	1.13
9	Islamic Azad University	47	0.04	1.11
10	Tongji University	46	0.02	1.09

**Table 4:** Top 20 keywords in scientific papers on fly ash-based geopolymer.

Ranking	Frequency	Centrality	Keywords
1	2689	0.02	fly ash
2	1191	0.03	mechanical property
3	1153	0.02	compressive strength
4	926	0.02	concrete
5	903	0.01	strength
6	836	0.02	cement
7	695	0.01	microstructure
8	655	0.01	performance
9	606	0.02	behavior
10	562	0.02	metakaolin
11	494	0.01	geopolymer concrete
12	489	0.02	geopolymer
13	426	0.03	temperature
14	421	0.01	slag
15	409	0.03	blast furnace slag
16	374	0.01	durability
17	320	0.01	Portland cement
18	298	0	mortar
19	260	0.01	workability
20	256	0.01	resistance

## Data Availability

The data presented in this study are available on request from the corresponding author upon reasonable request.

## Supplementary Materials

The following supporting information can be downloaded at: https://www.mdpi.com/article/10.3390/ma15144777/s1, Figure S1: Keywords with the strongest citation burst; Figure S2: Cited journals with the strongest citation bursts; Table S1: Distribution of scientific publications of fly ash-based geopolymer in WoS database between 1998–2022; Table S2: Top 20 categories in the field of fly ash-based geopolymer in 1998–2022; Table S3: Top 20 cited authors in scientific publications on fly ash-based geopolymer; Table S4: Top 20 cited journals in scientific publications on fly ash-based geopolymer; Table S5: Top 20 cited references in scientific publications on fly ash-based geopolymer.
