# Supplementary material for: A Bibliometric Analysis of Research Progress and Trends on Fly Ash-Based Geopolymer
Source: Materials (Basel). 2022 Jul 7;15(14):4777. doi: 10.3390/ma15144777 (PMC9324275; doi:10.3390/ma15144777)
Supplement: Supplementary file 1 [file materials-15-04777-s001.zip › materials-1771734-supplementary.pdf]

A bibliometric analysis of research progress and trends on fly ash-based geopolymers

Quanzhi Tian [a], Yinhai Pan [a, b], Yingchu Bai [a, b], Shuo Yao [a, b], Shiqiang Sun [c] *

[a] National Engineering Research Center of Coal Preparation and Purification, China University of Mining and Technology, Xuzhou 221116, China

[b] School of Chemical Engineering and Technology, China University of Mining and Technology, Xuzhou 221116, China

[c] School of Environmental Science and Engineering, Tongji University, Shanghai 200092, China

* Corresponding author

E-mail: cumtssq@163.com (S. Sun)

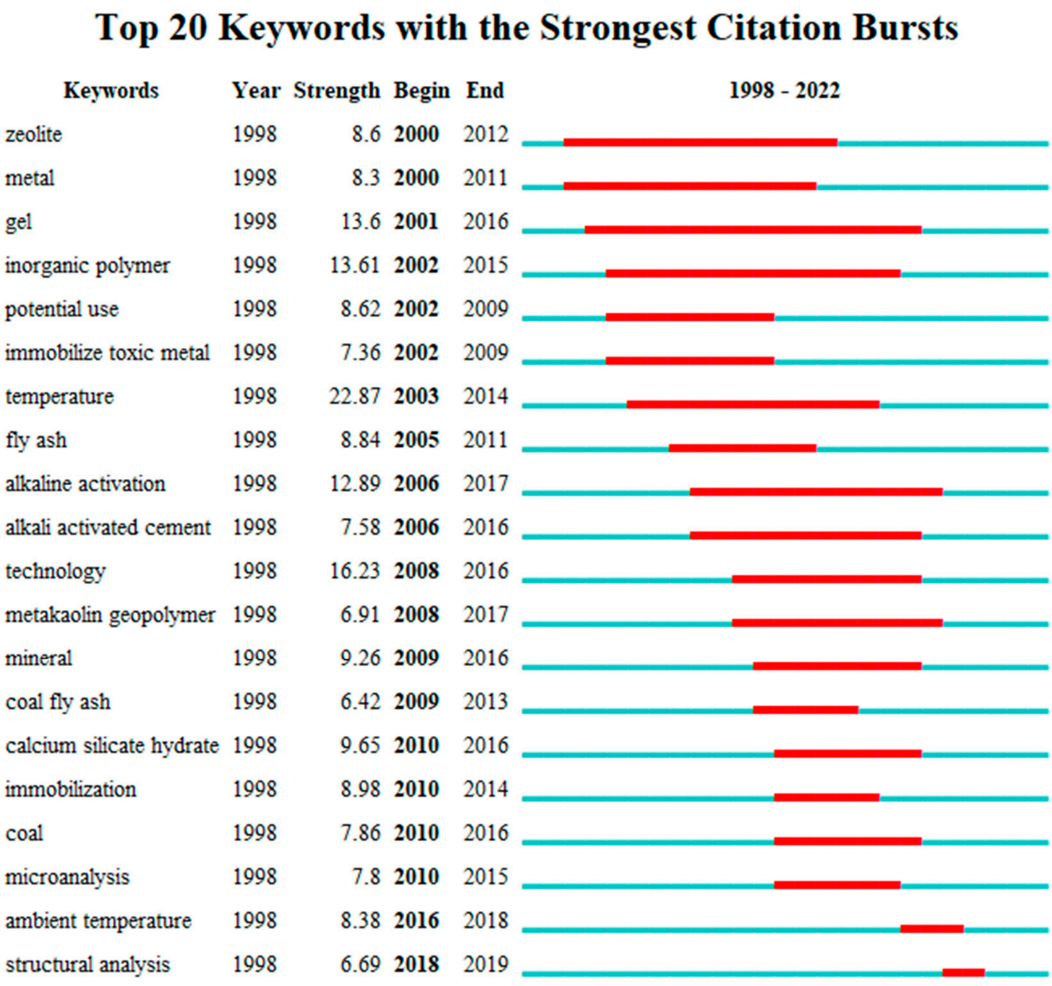

Figure S1. Strongest citation burst of keywords analysis.

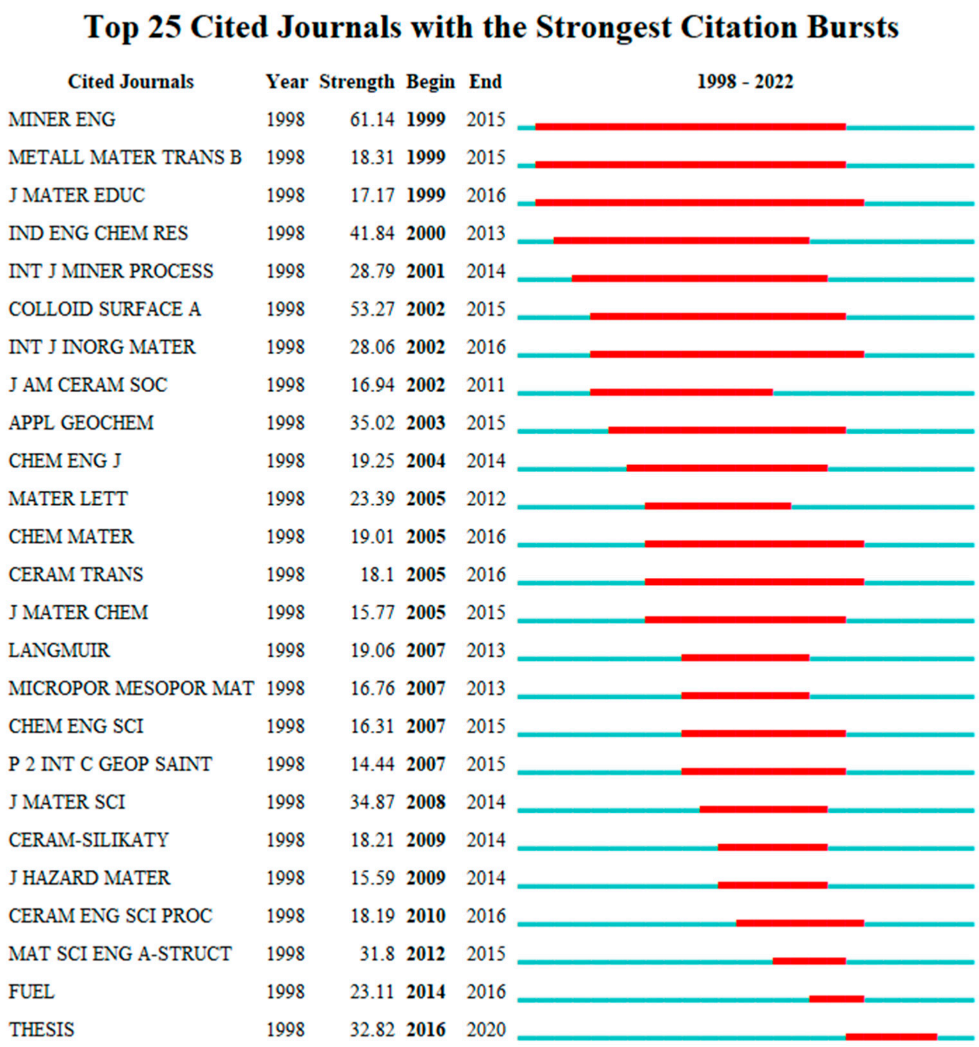

Figure S2. Strongest citation burst of journal co-citation analysis.

Table S1. Distribution of scientific publications of fly ash-based geopolymer in WoS database between 1998-2022.

**Year**	**Total publications**	**Proportion (%)**
1998	1	0.02
1999	1	0.02
2000	1	0.02
2001	2	0.05
2002	6	0.14
2003	4	0.10
2004	8	0.19
2005	11	0.26
2006	8	0.19
2007	27	0.65
2008	19	0.45
2009	35	0.84
2010	47	1.12
2011	54	1.29
2012	83	1.98
2013	128	3.06
2014	157	3.75
2015	189	4.52
2016	262	6.26
2017	303	7.24
2018	440	10.52
2019	514	12.29
2020	709	16.95
2021	915	21.87
2022	259	6.19

Table S2. Top 20 categories in the field of fly ash-based geopolymer in 1998-2022.

Ranking	Category	Count	Contribution (%)
1	MATERIALS SCIENCE	2507	16.00
2	ENGINEERING	2085	13.31
3	CONSTRUCTION & BUILDING TECHNOLOGY	1646	10.50
4	MATERIALS SCIENCE, MULTIDISCIPLINARY WE SCIENCE CI	1640	10.47
5	ENGINEERING, CIVIL	1223	7.81
6	ENVIRONMENTAL SCIENCES & ECOLOGY	468	2.99
7	CHEMISTRY	445	2.84
8	ENVIRONMENTAL SCIENCES WE SCIENCE CITATION INDEX E	407	2.60
9	MATERIALS SCIENCE, MULTIDISCIPLINARY	386	2.46
10	SCIENCE & TECHNOLOGY - OTHER TOPICS	381	2.43
11	ENGINEERING, ENVIRONMENTAL	338	2.16
12	PHYSICS	290	1.85
13	CHEMISTRY, PHYSICAL	288	1.84
14	GREEN & SUSTAINABLE SCIENCE & TECHNOLOGY	278	1.77
15	METALLURGY & METALLURGICAL ENGINEERING	259	1.65
16	MATERIALS SCIENCE, CERAMICS WE SCIENCE CITATION IN	242	1.54
17	ENGINEERING, CIVIL WE SCIENCE CITATION INDEX EXPAN	237	1.51
18	PHYSICS, CONDENSED MATTER WE SCIENCE CITATION INDE	198	1.26
19	PHYSICS, APPLIED	193	1.23
20	MATERIALS SCIENCE, COMPOSITES WE SCIENCE CITATION	190	1.21

Table S3. Top 20 cited authors in scientific publications on fly ash-based geopolymer.

**Ranking**	**Cited Authors**	**Count**	**Centrality**	**Contribution (%)**
1	Davidovits J	1943	0.15	6.83
2	Duxson P	1732	0.06	6.09
3	PROVIS JL	1146	0.1	4.03
4	Fernandez-Jimenez A	1003	0.1	3.53
5	PALOMO A	919	0.04	3.23
6	Bakharev T	899	0.13	3.16
7	Chindaprasirt P	861	0.03	3.03
8	Hardjito D	686	0.11	2.41
9	Xu H	677	0.06	2.38
10	Zhang ZH	641	0.01	2.25
11	Temuujin J	617	0.01	2.17
12	Van Jaarsveld JGS	581	0.07	2.04
13	Bernal SA	580	0.01	2.04
14	ASTM	545	0	1.92
15	Puertas F	520	0.05	1.83
16	Pacheco-Torgal F	514	0.03	1.81
17	Lee WKW	486	0.11	1.71
18	Nath P	482	0.01	1.69
19	YIP CK	450	0.08	1.58
20	Criado M	434	0.02	1.53

Table S4. Top 20 cited journals in scientific publications on fly ash-based geopolymer.

Ranking	Cited Journals	Count	Year	Centrality
1	CONSTR BUILD MATER	3261	2009	0.05
2	CEMENT CONCRETE RES	3209	2000	0.07
3	CEMENT CONCRETE COMP	2668	2004	0.11
4	J MATER SCI	2277	2000	0.03
5	J CLEAN PROD	1912	2012	0.01
6	J HAZARD MATER	1638	2005	0.03
7	CERAM INT	1580	2007	0
8	MATER DESIGN	1465	2011	0.02
9	FUEL	1350	2000	0.02
10	APPL CLAY SCI	1261	2009	0.03
11	J AM CERAM SOC	1231	2000	0.01
12	J MATER CIVIL ENG	1212	2005	0.02
13	MATER STRUCT	1192	2005	0.02
14	MINER ENG	1093	1999	0.01
15	COLLOID SURFACE A	1069	2000	0.06
16	J THERM ANAL	979	2000	0.01
17	ACI MATER J	956	2004	0.05
18	COMPOS PART B-ENG	914	2013	0
19	WASTE MANAGE	841	2000	0.01
20	MATER LETT	825	2003	0.07

Table S5. Top 20 cited references in scientific publications on fly ash-based geopolymer.

Ranking	Count	Centrality	Year	Reference
1	157	0.03	2015	Singh B, 2015, CONSTR BUILD MATER, V85, P78, DOI 10.1016/j.conbuildmat.2015.03.036
2	150	0.02	2016	Zhuang XY, 2016, J CLEAN PROD, V125, P253, DOI 10.1016/j.jclepro.2016.03.019
3	148	0.02	2018	Luukkonen T, 2018, CEMENT CONCRETE RES, V103, P21, DOI 10.1016/j.cemconres.2017.10.001
4	139	0.08	2014	Nath P, 2014, CONSTR BUILD MATER, V66, P163, DOI 10.1016/j.conbuildmat.2014.05.080
5	125	0.05	2014	Ismail I, 2014, CEMENT CONCRETE COMP, V45, P125, DOI 10.1016/j.cemconcomp.2013.09.006
6	114	0.03	2015	Provis JL, 2015, CEMENT CONCRETE RES, V78, P110, DOI 10.1016/j.cemconres.2015.04.013
7	113	0.01	2014	Deb PS, 2014, MATER DESIGN, V62, P32, DOI 10.1016/j.matdes.2014.05.001
8	109	0.02	2018	Provis JL, 2018, CEMENT CONCRETE RES, V114, P40, DOI 10.1016/j.cemconres.2017.02.009
9	103	0.07	2011	Somna K, 2011, FUEL, V90, P2118, DOI 10.1016/j.fuel.2011.01.018
10	99	0	2013	Turner LK, 2013, CONSTR BUILD MATER, V43, P125, DOI 10.1016/j.conbuildmat.2013.01.023
11	96	0.01	2007	Duxson P, 2007, J MATER SCI, V42, P2917, DOI 10.1007/s10853-006-0637-z
12	94	0.02	2017	Nath P, 2017, CONSTR BUILD MATER, V130, P22, DOI 10.1016/j.conbuildmat.2016.11.034
13	91	0.02	2015	Ken PW, 2015, CONSTR BUILD MATER, V77, P370, DOI 10.1016/j.conbuildmat.2014.12.065
14	90	0.04	2013	Ryu GS, 2013, CONSTR BUILD MATER, V47, P409, DOI 10.1016/j.conbuildmat.2013.05.069
15	88	0.01	2018	Ma CK, 2018, CONSTR BUILD MATER, V186, P90, DOI 10.1016/j.conbuildmat.2018.07.111

16	87	0.03	2015	Nath P, 2015, CEMENT CONCRETE COMP, V55, P205, DOI 10.1016/j.cemconcomp.2014.08.008
17	74	0.03	2017	Albitar M, 2017, CONSTR BUILD MATER, V136, P374, DOI 10.1016/j.conbuildmat.2017.01.056
17	74	0.01	2014	Gorhan G, 2014, COMPOS PART B-ENG, V58, P371, DOI 10.1016/j.compositesb.2013.10.082
18	74	0.01	2014	Provis JL, 2014, ANNU REV MATER RES, V44, P299, DOI 10.1146/annurev-matsci-070813-113515
19	73	0.06	2014	Hanjitsuwan S, 2014, CEMENT CONCRETE COMP, V45, P9, DOI 10.1016/j.cemconcomp.2013.09.012
